# Ileocaecal region impaction by plum pits in a South African patient: A case report

**DOI:** 10.4102/sajr.v30i1.3571

**Published:** 2026-09-09

**Authors:** Mobby M. Muzamhindo, Pieter B. Kotze, Christelle Ackermann

**Affiliations:** 1 Department of Medical Imaging and Clinical Oncology, Faculty of Medicine and Health Sciences, Tygerberg Hospital, Stellenbosch University, Cape Town, South Africa

**Keywords:** plant seeds, ileocaecal impaction, CT, bezoar, case report, Africa

## Abstract

Multiple plum pit impaction at the ileocaecal junction is an under-recognised cause of right lower quadrant pathology in Africa. A 54-year-old man presented with chronic abdominal pain, haematemesis and peritonism. CT confirmed partial luminal impaction with secondary appendiceal region inflammation. Conservative management was successful. Dietary history, careful plain radiograph review and CT with volumetric reconstruction were key diagnostic steps in this unusual presentation.

**Contribution:**

This case documents an uncommon occurrence of ileocaecal impaction from multiple plum pits with secondary appendiceal region inflammation, while highlighting volumetric CT reconstruction as a valuable diagnostic adjunct in confirming pit morphology.

## Introduction

Mechanical small bowel obstruction from ingested plant seeds is a rare clinical phenomenon, with fruit pits representing an infrequent, yet recognised aetiology.^1^ Although most ingested seeds pass through the gastrointestinal tract uneventfully, those with sharp morphology or substantial dimensions may become impacted at anatomically narrow sites, particularly within the ileocaecal region.^2,3^ The diagnostic imaging of such obstructions is challenging due to the varied radiodensity of plant seeds, although CT scanning has proven vital in identifying these intraluminal objects.^4^

There is notable geographic variability in the published literature on bowel obstruction due to plant seeds; the preponderance of documented cases originates from the Middle East, Asia and North America, with emerging reports from Africa.^3,4,5,6,7,8,9,10,11,12,13,14,15,16^ This observed geographic variability may be due to underlying cultural and dietary practices,^5^ as well as regional differences in medical publishing culture, case documentation and access to diagnostic imaging. This report seeks to augment the current understanding of this uncommon aetiology in an underreported demographic by presenting a South African patient with plum pit impaction at the ileocaecal region.

## Ethical considerations

An application for full ethical approval was made to the Health Research Ethics Committee, Stellenbosch University and ethics consent was received on 15 June 2026. The ethics approval number is C26/05/018 (Project ID 36504). Written informed consent was obtained from all individual participants involved in the study.

## Case presentation

A 54-year-old man presented with severe, worsening right-sided abdominal pain and reduced bowel frequency for 3 weeks, with an episode of haematemesis on the day prior to admission. On examination, he was peritonitic with rebound tenderness and guarding. Investigations were requested to exclude bowel perforation, bleed or mesenteric ischaemia. Initial full blood count revealed a mild leucocytosis of 12.3 × 10^9/L, which improved on repeat testing, with normal haemoglobin, platelets, C-reactive protein and electrolytes. These laboratory results were atypical for the patient’s clinical presentation, particularly given the severity of his abdominal pain and peritoneal signs, which typically correlate with more pronounced inflammatory markers.

Abdominal radiography demonstrated diffuse faecal loading without clear obstruction or perforation. Retrospective review identified subtle oval densities with central lucency in the right pelvis (Figure 1a and 1b). A prior radiograph 4 months earlier had shown non-specific faecal loading (Figure 1b). Subsequent CT (Figure 2a and 2b) demonstrated clusters of ovoid hyperdense structures with a hypodense centre in the terminal ileum, consistent with plum or apricot pits, some with air-filled cores. The terminal ileum was slightly dilated, and there was ileocolic mural thickening and oedema, indicating partial luminal impaction and local inflammatory change. Additionally, CT revealed a dilated, oedematous appendix without perforation or periappendiceal collection, with an initial diagnosis of uncomplicated appendicitis. There were no gallstones, and no gallbladder perforation or pneumobilia to suggest gallstone ileus.

**FIGURE 1:**
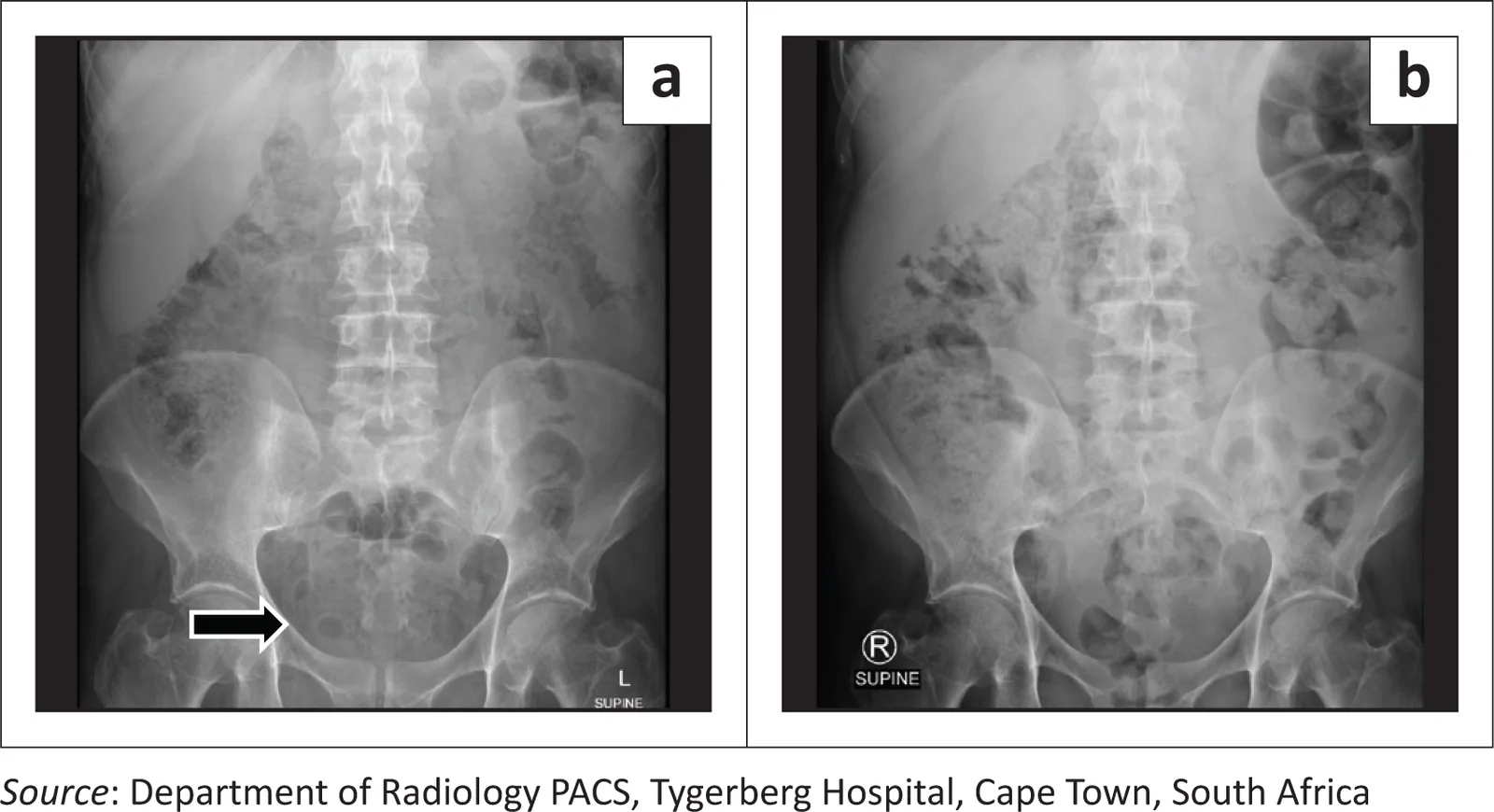
(a) Abdominal radiograph on day of admission revealing a non-specific bowel gas pattern with faecal loading. Retrospective review revealed oval masses with a radiodense rim in the right pelvis (arrow); (b) Prior abdominal radiograph showing diffuse faecal loading 4 months earlier. No evidence of radiodense foreign bodies.

**FIGURE 2:**
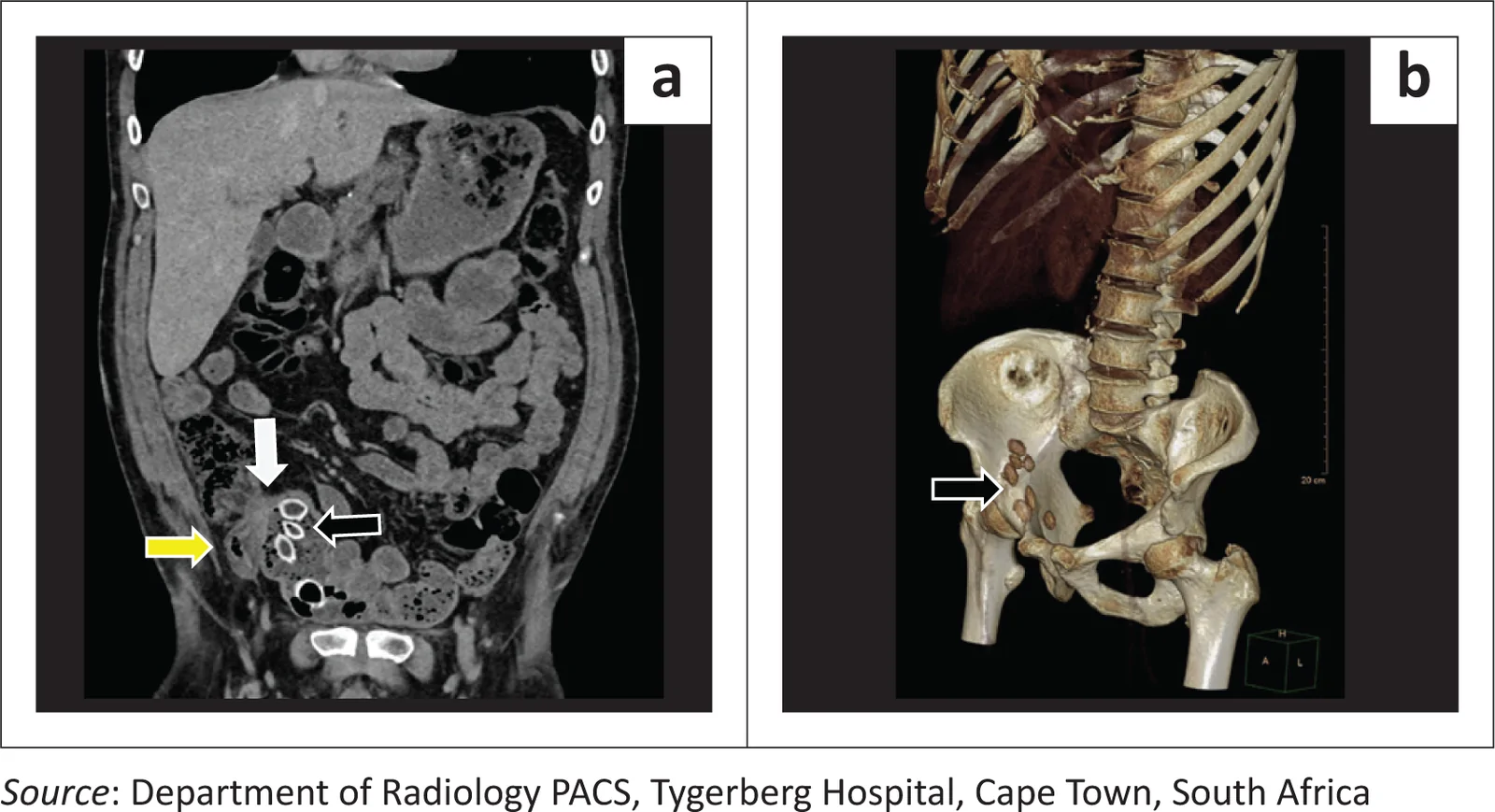
(a) Coronal CT of the abdomen. Clusters of oval hyperdense structures with central hypodensity representing the plum pits (black arrow). The terminal ileum is dilated with thickening of the ileal wall (white arrow). The appendix appears thickened and oedematous (yellow arrow); however, no appendicolith or pit was identified in this location; (b) CT volumetric reconstruction. Multiple oval structures seen in the right pelvis with the typical morphology of fruit pits (black arrow).

Conservative management with fluids and analgesia was initiated. Re-interview revealed chronic abdominal discomfort that acutely worsened following the ingestion of numerous plums, although the patient did not recall swallowing the pits. Additionally, he disclosed a prior appendicectomy, confirmed in the patient’s clinical past surgical notes but absent on the radiology request. Diagnostic laparoscopy was performed because he had dull left iliac fossa pain. This revealed extensive adhesions; however, no appendix was seen. The right iliac fossa inflammatory changes were therefore attributable to neighbourhood inflammation from the impacted pits, as laparoscopy excluded uncomplicated appendicitis. Gastroscopy also revealed gastritis, which may explain the patient’s haematemesis, for which proton pump inhibitors had been administered. The patient eventually passed the plum pits successfully. No further imaging was performed. He was discharged on postoperative day 3, following an uncomplicated recovery, for outpatient follow-up.

## Discussion

Small bowel obstruction is commonly caused by adhesions or hernias^7^; less common aetiologies include malignancy, inflammatory bowel disease or ingested foreign bodies, such as phytobezoars, which are concretions of undigested food.^16^ Plant seeds, particularly fruit pits, sometimes form bezoars; however, intestinal obstruction from plant seeds is a distinct clinical subcategory and less commonly reported.^5,16^ Plant seeds frequently pass through the gastrointestinal tract uneventfully because of their small size. However, if the seeds are of considerable size or with sharp edges, they may become lodged in the intestinal tract, particularly when existing gastrointestinal pathology is present.^2,3,13^ Notably, plum species (including *Prunus africana*) have recognised ethnomedicinal uses in Africa for gastrointestinal conditions,^17^ which may contribute to the ingestion of pits by some patients.

In the compiled literature, the anorectum remains the most common site of impaction (approximately 64% of 172 cases), while the terminal ileum or ileocaecal region is relatively uncommon, accounting for only about 12% of reported cases.^1,2,3,4,5,10,11,12,13,14,15,16,18,19^ Among the cases that reported terminal ileum impaction, the majority (62%) reported impaction from multiple pits; however, none involved plum pits.^1,5,10,11^ In the reviewed literature, plum pits were only reported as single seeds in the proximal small bowel.^12,13^ This case is therefore a noteworthy presentation of ileocaecal impaction caused by multiple plum pits, the risk possibly increased by prior surgery in the ileocaecal region. The ethnomedicinal use of plum fruits raises the plausible hypothesis that the patient may have ingested multiple plums as a remedy for his chronic constipation.

Most cases described bowel obstruction as the most common complication.^1,2,3,4,5,10,13,14,16,19^ A few cases documented perforation, intussusception, pseudodiverticulum of the terminal ileum and rectal wall thickening.^5,9,11,12,15,20^ While the presented case did not have overt imaging features of obstruction, terminal ileal thickening was noted, similar to the findings of Sinagra et al.^18^ Regional neighbourhood appendiceal inflammation is also known to occur with impacted foreign bodies or other ileocaecal pathology.^5^

The imaging appearances of ingested fruit pits and plant seeds vary by species in size, shape, density and internal architecture. On radiography, seeds may present as subtle radiopaque intraluminal densities or remain occult.^5,6,20^ On CT, they typically appear as ovoid or elongated hyperdense structures with a hypodense or air-filled centre.^4,12,13,15^ Larger seeds such as durian and mango are more conspicuous,^3,14^ while smaller seeds such as sunflower or grape pips may be easily overlooked,^4,10^ and clustered seeds can mimic a bezoar rather than discrete foreign bodies.^10^ Bone window and multiplanar reformatting are recommended practices to aid accurate diagnosis.^15^ Additionally, volumetric reconstruction can improve morphological characterisation and diagnostic confidence. Familiarity with the morphological appearance of common fruit pits and plant seeds is essential for accurate aetiological identification.

The differential diagnoses included mechanical obstruction (such as gallstone ileus or adhesion-related small bowel obstruction), malignancy (such as right-sided colon cancer) and inflammatory conditions (such as ileocaecal tuberculosis or Crohn’s disease). While hyperdense lesions at the ileocaecal junction can mimic gallstone ileus,^14^ the absence of pneumobilia or biliary-enteric fistula, and the characteristic morphology of fruit pits, favoured mechanical impaction. Despite the patient’s surgical history and adhesive bands on laparoscopy, a non-obstructive bowel gas pattern excluded adhesion-related small bowel obstruction. Similarly, ileocaecal tuberculosis and Crohn’s disease – which typically manifest with mural thickening and localised inflammation^15^ – were excluded by the absence of necrotic lymphadenopathy, ascites, collections, skip lesions, creeping fat and engorged vasa recta. Furthermore, right-sided malignancy was ruled out by the absence of a discrete circumferential or eccentric enhancing soft tissue bowel mass or regional lymphadenopathy.

The patient presented with atypical abdominal symptoms. Haematemesis was likely an incidental finding attributed to the gastritis identified on gastroscopy. Given the patient’s prior total appendicectomy, the appendiceal region inflammation indicated inflammatory sequelae from partial luminal impaction by the plum pits rather than stump appendicitis. Radiologists and physicians should consider fruit pit impaction in the differential diagnosis of atypical right lower quadrant disease.

## Conclusion

Fruit pit impaction should be included in the differential diagnosis for atypical right lower quadrant presentations, with intentional dietary history elicitation, as this is often not volunteered by patients. Plain radiographs should be scrutinised for subtle densities, while CT volumetric reconstruction can further improve the detection of fruit pits, in addition to bone windows and multiplanar reformatting.
